# More than skin deep: about the influence of self-relevant avatars on inhibitory control

**DOI:** 10.1186/s41235-022-00384-8

**Published:** 2022-04-08

**Authors:** Maximilian A. Friehs, Martin Dechant, Sarah Schäfer, Regan L. Mandryk

**Affiliations:** 1 The Interaction Lab, Department of Computer Science, University of Saskatchewan, Saskatoon, Canada; 2 School of Psychology, University College Dublin, Dublin, Ireland; 3 Department of General Psychology and Methodology, University of Trier, Trier, Germany

**Keywords:** Response inhibition, Stop-signal game, Avatar identification, Self-prioritization effect

## Abstract

One important aspect of cognitive control is the ability to stop a response in progress and motivational aspects, such as self-relevance, which may be able to influence this ability. We test the influence of self-relevance on stopping specifically if increased self-relevance enhances reactive response inhibition. We measured stopping capabilities using a gamified version of the stop-signal paradigm. Self-relevance was manipulated by allowing participants to customize their game avatar (Experiment 1) or by introducing a premade, self-referential avatar (Experiment 2). Both methods create a motivational pull that has been shown to increase motivation and identification. Each participant completed one block of trials with enhanced self-relevance and one block without enhanced self-relevance, with block order counterbalanced. In both experiments, the manipulation of self-relevance was effective in a majority of participants as indicated by self-report on the Player-Identification-Scale, and the effect was strongest in participants that completed the self-relevance block first. In those participants, the degree of subjectively experienced that self-relevance was associated with improvement in stopping performance over the course of the experiment. These results indicate that increasing the degree to which people identify with a cognitive task may induce them to exert greater, reactive inhibitory control. Consequently, self-relevant avatars may be used when an increase in commitment is desirable such as in therapeutic or training settings.

## Public significance statement

The present research suggests that self-relevant avatars can increase identification, and this increased identification is predictive of an increase in response inhibition speed under certain conditions. This was replicated in two independent but conceptually similar studies. These results bear implications for training, therapy, game design and everyday life alike. For example, increasing avatar identification and self-relevance might be simple ways to increase a training effect and fuel positive therapeutic outcomes. Further, in video games (especially free-to-play titles), avatar customizations, at least cosmetic ones, are often locked behind a paywall and since they do not provide a power-boost for the character in game, they are often assumed to not matter for in-game performance. However, our results do suggest that being able to express yourself in the game and identifying with the avatar may be important for performance

## Introduction

### Reactive response inhibition: importance in daily life and measurements

Stopping an already initiated response is vital for adaptive everyday behavior. For example, a basketball player might have to cancel their jump once they recognize the opponent is going for a pump-fake instead of a basket throw. This ability can be measured using tasks such as the stop-signal task (SST) (Lappin & Eriksen, 1966; Logan et al., 1984; Verbruggen & Logan, 2008; Verbruggen et al., 2019). The typical SST presents participants with a go-signal to which a response has to be made. Ordinarily left and right pointing arrows are used to elicit the press of the left or right arrow key. Usually, in 25% of all trials, a stop-signal appears after the initial go-stimulus onset. After the stop-signal participants have to withhold the already initiated go-reaction. Recently, the stop-signal game (SSG) was developed and validated as an alternative gamified version to the ordinary SST (Friehs et al., 2020). The SSG is conceptually identical to the ordinary SST and reliably measures response inhibition, but importantly, the subjective enjoyment for the SSG is rated higher compared to the ordinary SST. Further, the addition of several game-like and aesthetic elements allows for additional customization (e.g., changes in the avatar’s appearance or the surroundings)—which could be used to induce motivation—to a degree that is not possible with the ordinary SST.

### Modifying response inhibition performance

Evidence suggests that performance on a stop-signal task is influenced by internal state changes; for example, reactive response inhibition can be altered due to changes in neurological states using noninvasive brain stimulation (Friehs, Brauner, et al., 2021; Friehs, Frings, et al., 2021; Friehs & Frings, 2018, 2019; for a review see Friehs, Brauner, et al., 2021; Friehs, Frings, et al., 2021) or after intense training (Kramer, Hahn, Cohen, & al., 1999; Tsai, 2009). Little research has been done on motivational influences on response inhibition, but it would make intuitive sense that a change in motivation due to self-relevance can impact performance (Schall & Godlove, 2012). Consequently, the studies in this manuscript set out to test whether the ability to inhibit inappropriate responses depends on how *self-relevant* stimuli are.

### Increased motivation through self-referential avatars

This research focused on how self-relevance may influence response inhibition. The self-relevance of stimuli was manipulated using the self-prioritization effect (SPE) and the avatar identification effect (AIE). The AIE paradigm calls for participants to customize the appearance, personality, and attributes of an avatar in such a way that this avatar represents themselves. Previous research demonstrates that this leads to increased avatar identification, which in turn has been shown to increase motivation, reduce attrition, increase enjoyment, and improve the efficacy of cognitive training (Birk & Mandryk, 2018, 2019; Birk, Atkins, et al., 2016; Birk, Mandryk, et al., 2016; Birk, Mandryk, & Atkins, 2016). Avatar customization gives the player control, fosters a sense of autonomy, and makes the player feel as if they have more impact on the task (Sundar & Marathe, 2010). Birk and Mandryk (2019) demonstrate that in attentional-bias modification training aimed at enhancing mood regulation by shifting attention away from negative stimuli, avatar customization increased the training’s effectiveness. Specifically, avatar customization enhanced resilience to negative mood inductions after the training.

However, if customization is not an option (e.g., due to technical or task-based restrictions), self-relevance can still be achieved by making use of the SPE. The SPE describes the association of a formerly neutral stimulus with the self, which has been shown to improve performance in various tasks (Constable et al., 2019; Golubickis et al., 2018; Schäfer et al., 2020; Sui et al., 2012). Here, it has been demonstrated that avatars can be associated with the self, and the avatar will then be prioritized (Mattan et al., 2017; Mattan et al., 2015; Friehs, Schäfer & Frings, *under review*). For example, a recent study by Friehs, Schäfer & Frings (*under review*) revealed that people demonstrate enhanced processing for stimuli related to their own avatar and that self-associations are stronger if the to-be-associated stimuli are closer to the avatar’s upper torso—suggesting a projected location of the self in the avatar. This is in line with previous studies suggesting that the place of the self is perceived to be centered around the upper torso or head area (Limanowski & Hecht, 2011). These results imply that stimuli are processed preferentially when presented close to the self-relevant avatar (specifically, close to the projected ego-center of the avatar).

### Customization and self-relevance in games

Although the ability to change and adapt a playable avatar is a feature of many games, some games have also incorporated cosmetic avatar changes into their business model. For example, Riot games adopted the business practice of releasing cosmetic in-game items designed by the winner of their yearly world championship tournament (see also Hamari & Lehdonvirta, 2010; Musabirov et al., 2017; Oh & Ryu, 2007). It is generally assumed that cosmetic changes, such as a new avatar outfit, do not affect core gameplay mechanics, and thus have no impact on gameplay or game balance. However, many players invest a lot of time and money into customizing their avatar in games, which in turn may further increase the self-identification with the avatar and the motivation to play the game (Bessière et al., 2007; Qiu et al., 2021). This proposition is in line with research demonstrating that among other things, character dedication, social distinction, and self-gratification can be driving factors that lead players to purchase a cosmetic in-game item (Lehdonvirta, 2009; Marder et al., 2019). However, so far it remains unclear how self-relevance affects in-game performance.

### The current studies: the relation of self-relevance through Avatar identification and performance

The influence of self-relevance on response inhibition was tested in two studies. We manipulated the self-relevance of the avatar and measured the effect of this identification increase in performance. To test the postulated effect reliably, we manipulated identification increase in two different ways: by customizing the avatar in the first study and by assigning an avatar representing the participant in the second study. Note that the second study does not match the avatar appearance to the person but assigns an avatar as self-relevant and uses the SPE to induce the association with the self.

In both studies, SSG performance was measured twice: once with an avatar with enhanced self-relevance and once with a standard avatar. We hypothesize that the increased connection with the given avatar in comparison with either a non-customized, generic avatar (Exp. 1) or a non-self-associated (here: stranger-associated) avatar (Exp. 2) increases self-reported identification. Further, we hypothesize that if participants do show a stronger identification with the self-relevant avatar, their performance will increase. Hence, in both studies, we postulate increased performance scores in the SSG in the condition with the high-identification avatar in comparison to the condition with the low-identification avatar.

## Experiment 1: use of avatar customization

When applying the AIE, participants show higher intrinsic motivation, more need satisfaction, and higher performance after being able to customize their avatar (Birk, Atkins, et al., 2016; Birk, Mandryk, et al., 2016; Birk, Mandryk, et al., 2016; Birk & Mandryk, 2018; Birk & Mandryk, 2019). Thus, in this study, participants run through the SSG twice: once with a customized avatar and once with a preset avatar. The order of the two SSG blocks was counterbalanced across subjects. A participant’s performance with a self-made, customized avatar was compared to their performance with a generic, preset avatar.

### Method

#### Sample

We recruited participants over Amazon Mechanical Turk (MTurk) and recorded 71 complete datasets (28 female, 43 male, mean age = 37.92, SD = 11.55). Based on previous research on the AIE (Birk, Atkins, et al., 2016; Birk, Mandryk, et al., 2016), we expected a medium-sized effect of *f* = 0.35 as well as a medium-sized correlation between repeated performance measures of *r* = 0.4 (Friehs & Frings, 2018, 2019a; Friehs et al., 2020; Friehs et al., 2020; Friehs et al., 2020c; Friehs, Brauner, et al., 2021; Friehs, Frings, et al., 2021). Together with an α-value of 0.05 and a power of 1 – β = 0.95, a sample of at least 30 participants was planned (power analysis was carried out using G*Power 3.1.3; Faul, Erdfelder, Lang, & Buchner, 2007). To test also for smaller effects, as well as to potentially deal with interaction effects with the (between-subject) factor of the order of the SSG parts (see above), we collected data from a larger sample. Participants were monetarily compensated for their participation (12$/h) and the whole study took 50 min to complete. The study was approved by the local ethics committee. All participants provided written informed consent.

#### Design

The study had a repeated-measures design to evaluate the effect of avatar identification on performance within participants. Thus, we used a 2 (avatar identification: *high* vs. *low*) × 2 (order: *high identification first* vs. *low identification first)* mixed design, with the order being counterbalanced between participants. The main dependent variable was the stop-signal reaction time (SSRT, i.e., the estimate of time needed to respond to the stop-signal and to cancel the movement), which is a measure of the reactive inhibition process.

#### Procedure

Participants were briefed about their tasks in the study, and afterward, participants were either tasked with customizing one avatar to make it resemble their own character (high-identification condition) or were provided with a generic (low-identification condition).

**Fig. 1:**
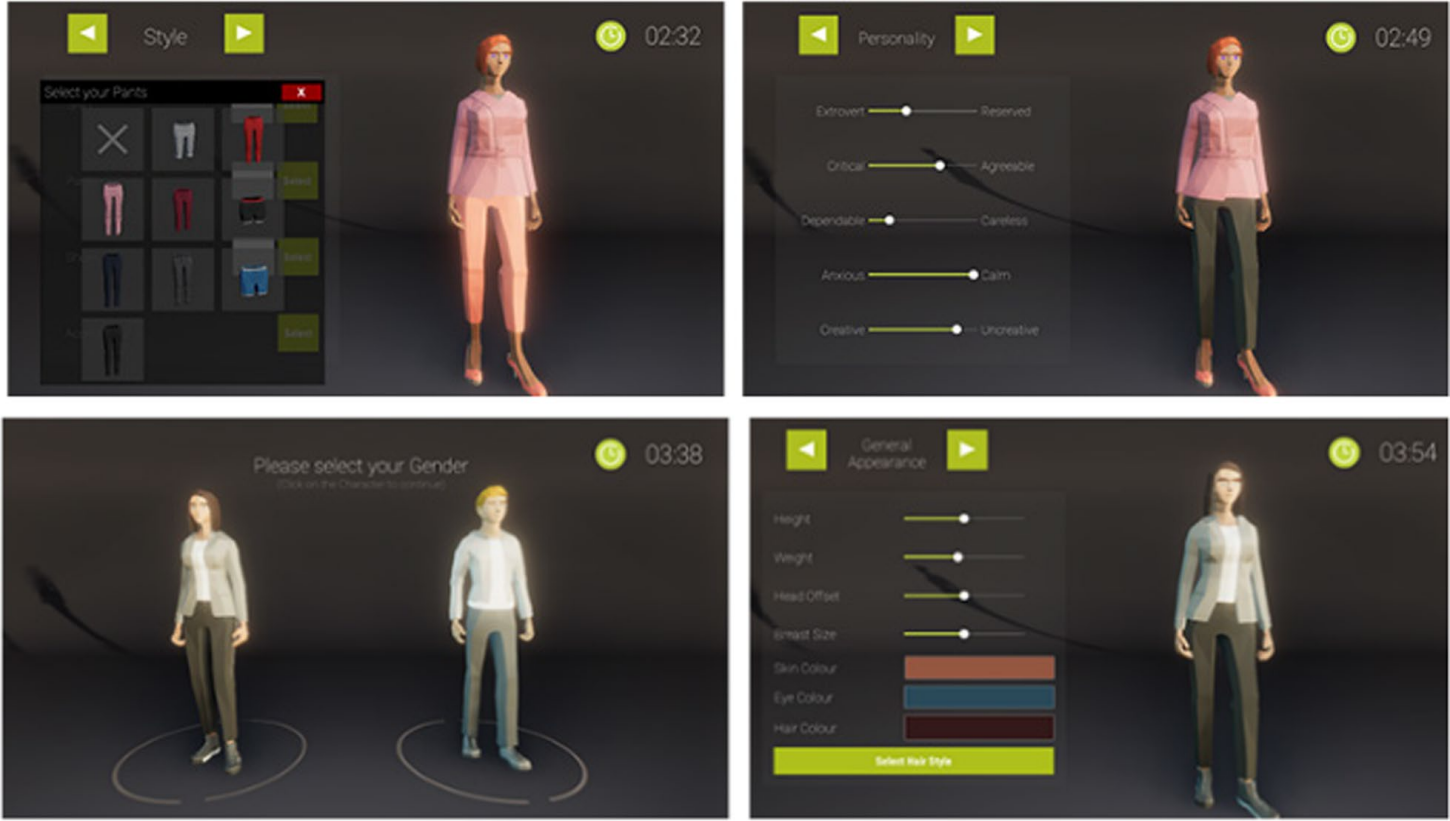
Character Editor. Participants were able to customize their character’s clothing (top left), personality (top right), body type (bottom left) and physical attributes as well as body proportions (bottom right). The character was imported into the game, but neither the visual nor personality features of the customized avatar had any impact on the task

In the customized condition, participants could adjust the appearance and personality of their avatar. Possible customizations included gender, height, weight, muscles, heads, the shape of facial features, chest size and the color of the skin, eyes, hair and beard. Further, the participant could choose the style and color of the avatar’s clothing, as well as add accessories such as glasses headphones, or hats. Furthermore, participants were asked to describe the personality of their avatar by manipulating five 7-point scales, which each described one personality trait, based on the 10-item short version of the Big Five inventory (BFI-10) (Rammstedt et al., 2013). Figure 1 shows the different steps of the avatar editor.

Participants were asked to complete one of the two SSG conditions (high-identification or low-identification) with the accompanying avatar. Once the first SSG condition was finished, participants filled out the Player Identification Scale (PIS; Van Looy et al., 2012) to assess identification with the avatar. After that the second SSG condition, the PIS was presented a second time. Once both sessions were completed, participants answered demographic questions.

**Fig. 2:**
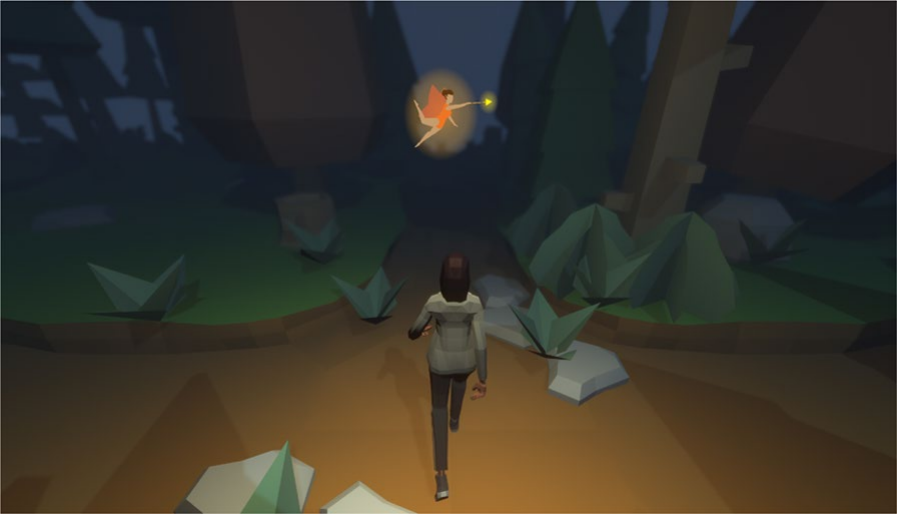
Stop-signal game. Participants had to navigate a character through an enchanted forest. At each intersection, after a variable inter-trial interval of 500–1500 ms, the fairy at the top of the screen indicated the direction in which the character should turn (here: right turn). An auditory stop-signal was presented on a subset of trials; after a stop-signal, participants were tasked to withhold their already initiated action

*Stop-Signal Game* We used a, previously validated, gamified version of the SST. Here, the game takes the form of an infinite runner. The game was implemented using the Unity3D engine (for technical details please refer to ). The underlying task architecture was based on the recommendations for the use of the SST by Verbruggen and colleagues (Verbruggen & Logan, 2008, 2009; Verbruggen et al., 2019). The SSG further presented the participants with a cover story that helped contextualize their performance. Specifically, participants were told they were lost in an enchanted forest and a fairy would help them escape by pointing either to the left or right at every crossroads. However, they were further told that a witch might attempt to take on the appearance of the fairy in order to trick the player into going deeper into the haunted woods. This witch, however, can be detected by an audio-cue. This audio cue serves as the stop-signal in the task. Figure 2 depicts the SSG.

Each session consisted of a total of 300 trials, containing 75% go- and 25% stop-trials. The 300 trials were divided into three blocks with a 15 s break in between. Participants were instructed to react as fast and accurately as possible to the go-stimulus (i.e., a fairy pointing left or right) with the left or right arrow key and withhold their reaction when a stop-signal (i.e., a noise presented over headphones) occurs. The go-stimulus was presented for a maximum of 1500 ms or until reaction. The stop-signal was played over the headphones following a variable delay (the stop-signal delay, SSD). The *SSD* represents the delay between the onset of the go- and the stop-signal and was initially set to 250 ms. The SSD was continuously adjusted with the staircase procedure to obtain a probability of responding of 50%. After the reaction was successfully stopped (i.e., button press was inhibited), the SSD was increased by 50 ms, whereas when the participants did not stop successfully, the SSD was decreased by 50 ms. The inter-trial interval was set to a random value between 500 and 1500 ms. Several different performance measures were logged and calculated including the SSD and the probability of making a (wrong) response when a stop-signal is presented (*p(response|signal)*). Furthermore, two variables that are directly related to accuracy were logged: first, the amount of *omission errors* (reflecting the probability of missed response on no-signal trials) and second, the *choice errors* (reflecting the probability of a wrong response on no-signal trials). Additionally, we logged two RT variables; *no-signal RT* reflects the speed of correct responses on trials without a stop-signal, and *signal RT*, which indicates the latency of the incorrectly executed response on stop-signal trials. Furthermore, the probability of a *correct inhibition* (i.e., the likelihood of inhibiting an already initiated action) was recorded for each participant. Most importantly, the stop-signal reaction time (SSRT) could be calculated based on a participant’s performance. The estimation of the SSRT was based on the integration method with replacement of omissions (for a detailed description please refer to Friehs et al., 2020a, 2020b; Verbruggen et al., 2013, 2019).

#### Player identification scale

The PIS was used to measure the participant’s identification with the avatar (Van Looy et al., 2012). The scale encapsulates three subscales: similarity identification, embodied identification, and wishful identification. Similarity identification is measured through items such as “My character is similar to me in many ways”, “I identify with my character” and “My character is an extension of myself”. Embodied identification is measured through items such as “When I am playing, it feels as if I am my character”, “In the game, it is as if I become one with my character” and “In the game, it is as if I act directly through my character”. Wishful identification is measured through items such as “If I could become like my character, I would”, “My character is an example to me” and “My character has characteristics that I would like to have”. Participants rated their agreement to different avatar-related statements on a 5-point scale from “strongly agree” to “strongly disagree”.

#### Analysis plan

Data Analysis was done in five phases: First, in the data reduction stage, any participant with faulty or invalid data as well as participants that made too many errors were removed from analysis. See *Data Reduction* section below for more details. Second, in a preliminary analysis stage, all pre-requisites for analysis were evaluated. This includes showing that there is a statistical difference between the average signal-response time and the average no-signal RT (for more details see Verbruggen et al., 2019) as well as reliability analysis for the player identification scale and its subscales. Third, as a form of manipulation check, we evaluated whether player identification differed between the custom and generic avatar condition. Fourth, overall performance was evaluated. Fifth, we investigated the hypothesis that player identification, as measured by the PIS, is predictive of a performance improvement in the SSG as indicated by a changed response inhibition. Specifically, the difference between avatar identification should be predictive of the SSRT difference between generic and custom avatar conditions.

#### Data reduction

Although MTurk data quality has been shown to be reliable in general, there are still task-specific exclusion criteria to be considered (Buhrmester et al., 2011; Chmielewski & Kucker, 2020). We followed the recommendations by Verbruggen and colleagues (Verbruggen et al., 2019; Verbruggen & Logan, 2015, see also Friehs, Brauner, et al., 2021; Friehs, Frings, et al., 2021). First, we tested the horse-race assumption for every participant by comparing signal-response reaction time (RT) and no-signal RT. The horse-race assumption states that SSRT can only reliably be estimated if the RT on unsuccessful stop trials is smaller than the mean go-RT. Second, participants were excluded if their p(response|signal) was smaller than 0.25 or larger than 0.75 in either session. Third, outliers were determined based on the Tukey outlier criterion (Tukey, 1977), and removed if the accuracy on go-trials was 3 or more standard deviations below the sample. Based on these criteria, eighteen participants had to be excluded, resulting in a final sample of 53 subjects (mean age = 39.1, SD = 12.1, 33 male and 20 female). Participants’ self-identified ethnicity was predominantly White (*n* = 40), with a minority of people identifying as Asian (*n* = 6), Black or African American (*n* = 5), American Indian or Alaskan (*n* = 1) or Hispanic/Latino (*n* = 1).

### Results

**Fig. 3:**
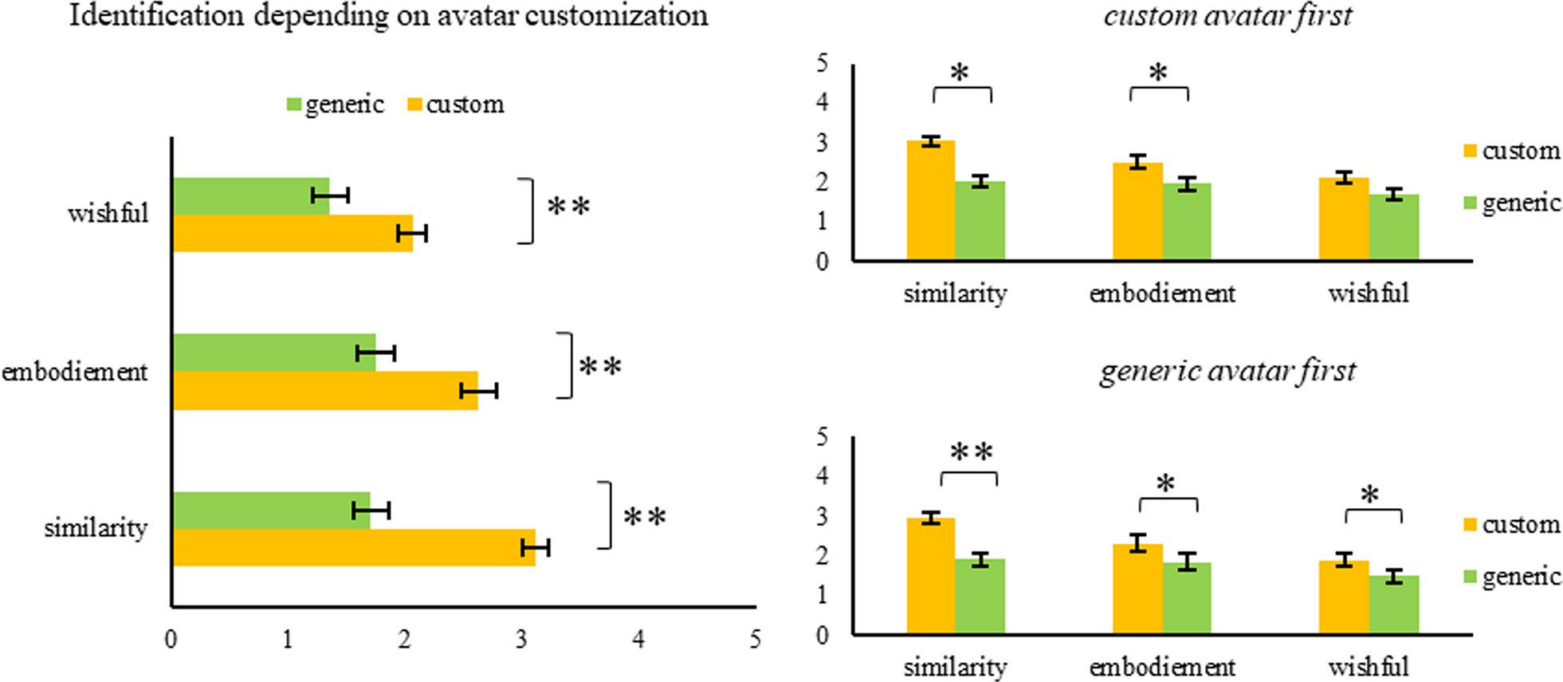
Player Identification Scale results for all valid participants, split by the order in which the SSG was played (top right: custom avatar first, bottom right: generic avatar first). *p < .05, **p < .001. Error bars signify the standard error of the mean

**Fig. 4:**
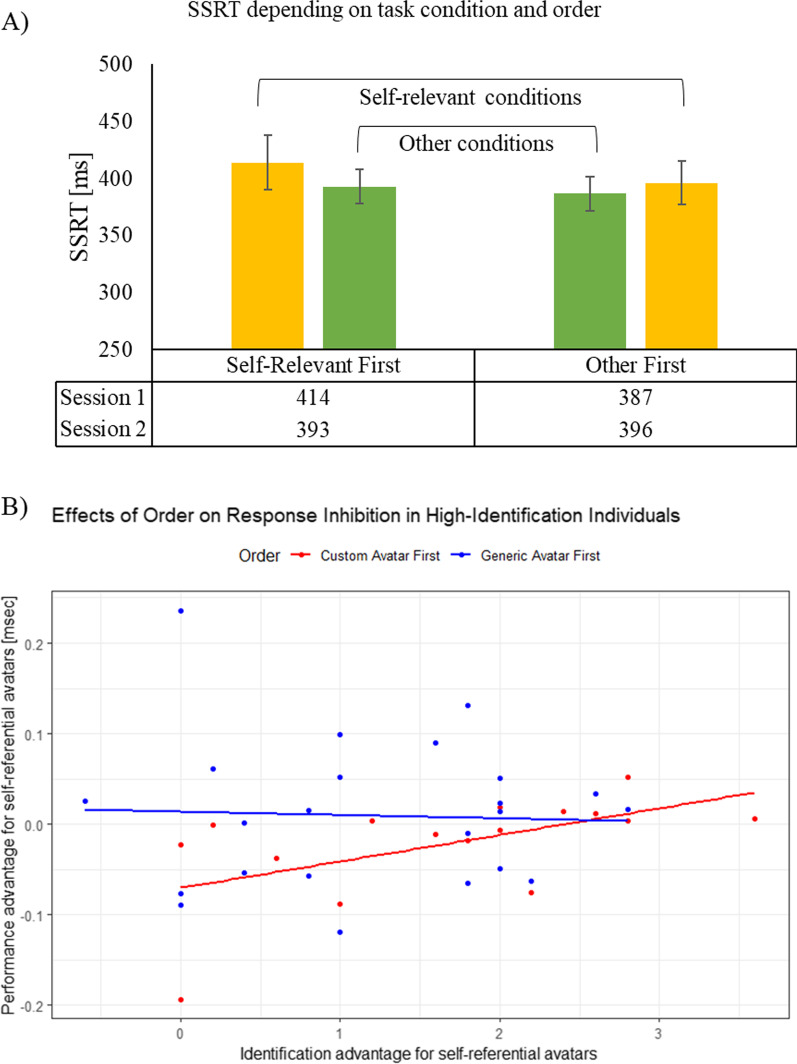
Only participants that identified with the self-relevant avatar (i.e., for whom the manipulation was successful) were considered for this analysis. Note that in the stop-signal paradigm, a lower SSRT indicates a better performance. A lower SSRT means that the estimated inhibition process is faster. **A** Descriptive statistics for both conditions depending on the task order in which they were completed. If participants played the self-relevant condition first, their SSRT (i.e., the response inhibition speed) improved from session 1–2; vice versa for the other order condition. Error bars depict the standard error of the mean. **B** Increased identification through similarity with the avatar is predictive of a performance change; as measured by the difference in SSRT between the two conditions. The higher identification with the self-relevant avatar, when it was used first, leads to a larger difference between the task conditions, which correspond to session 1 and 2. This performance difference is predicted by the strength of the identification with the avatar

The results show that the reactive inhibition process (as measured by SSRT) is affected by identification with the custom, participant-made avatar. Specifically, a performance increase over time was observed in participants with increased identification. For details on results, see Fig. 3 for the results on avatar identification and Fig. 4 for the performance change predicted by identification.

#### Preliminary SSG analysis

To validate the gathered data, it is recommended to show that there is a statistical difference between the average signal RT (i.e., RTs of correct responses during go-trials) and the average no-signal RT (i.e., RTs of false responses during stop-trials) for each experimental condition (Verbruggen & Logan, 2015; Verbruggen et al., 2019). We crossed the trial type (signal vs. no-signal) and the avatar identification (*high* vs. *low*) in a 2 × 2 ANOVA. Results show significantly different RTs between signal and no-signal trials as indicated by the significant main effect of trial type, *F*(1, 52) = 299.27, *p* < 0.001, η_p_^2^ = 0.85). No main effect of avatar identification and no interaction was found, *F*(1, 52) = 2.45, *p* = 0.12 and *F*(1, 52) = 2.98, *p* = 0.09, respectively. Together, these results demonstrate a valid SSG measurement.

#### Manipulation check

The PIS served as a manipulation check. Reliability scores (Cronbach’s alpha) for all PIS subscales were computed, split by the order on which participants went through the study procedure. Results show that Cronbach’s alpha was satisfactorily high across all subscales: Similarity identification (custom avatar first = 0.93, generic avatar first = 0.95), embodied identification (custom avatar first = 0.97, generic avatar first = 0.95), wishful identification (custom avatar first = 0.87, generic avatar first = 0.94). Hence, player identification was measured with high reliability. A 2 (avatar identification: *high* vs. *low*) × 2 (order: *high identification first* vs. *low identification first*) × 3 (subscale: *similarity* vs. *embodiment* vs. *wishful*) MANOVA with the PIS scores as the dependent variable was calculated. Most importantly, the main effect of avatar identification was significant, *F*(1, 51) = 24.37, *p* < 0.001, η_p_^2^ = 0.32, indicating higher identification with the avatar in the high-identification (i.e., custom avatar) condition compared to the in the low-identification condition. Moreover, the main effect of subscale was significant, *F*(2, 50) = 45.83, *p* < 0.001, *η*_p_^2^ = 0.65, indicating that scores at the similarity subscale were rated higher than those of the wishful or embodiment subscales. The main effect of order did not reach statistical significance, *F*(1, 51) = 2.53, *p* = 0.12. The two-way interaction of avatar identification and subscale was significant, *F*(2, 50) = 13.42, *p* < 0.001, *η*_p_^2^ = 0.35. This indicates that the difference between the two avatar identification conditions were different depending on the subscales. The two-way interactions of avatar identification and order as well as subscale and order were not significant, *F*(1, 51) = 0.05, *p* = 0.82 and *F*(2, 50) = 1.74, *p* = 0.19, respectively. The three-way interaction was also not significant, *F*(2, 50) = 0.50, *p* = 0.61.

#### Overall performance analysis

The main dependent variable in the SSG is the SSRT as an indicator of the speed of the reactive response inhibition process. However, a 2 (avatar identification: *high* vs. *low*) × 2 (order: *high identification first* vs. *low identification first*) ANOVA did not reveal an overall significant effects of avatar identification on SSRT and neither the effect of task order nor the interaction was significant; all *F*s < 2.45, all *p*s > 0.12. A comparable 2 (avatar identification: *high* vs. *low*) × 2 (order: *high identification first* vs. *low identification first*) ANOVA with the SSD as the dependent variable revealed two non-significant main effects of condition (*F*(1, 51) = 1.01, *p* = 0.32) and order (*F*(1, 51) = 1.27, *p* = 0.27), while the interaction (*F*(1, 51) = 15.08, *p* < 0.001, *η*_p_^2^ = 0.23) was statistically significant, which indicates a practice effect from session 1 to 2. The same data pattern was found in no-signal RTs (i.e., RTs of correct responses during go trials) as well as in signal RTs (i.e., RTs of false responses during stop trials). In both ANOVAs, a significant interaction was found, *F*(1, 51) = 11.75, *p* < 0.001, *η*_p_^2^ = 0.19 for no-signal RTs, and *F*(1, 51) = 9.67, *p* < 0.01, *η*_p_^2^ = 0.16 for signal RTs. However, no main effect of avatar identification, *F*(1, 51) = 3.03, *p* = 0.09 for no-signal RTs, and *F*(1, 51) = 1.07, *p* = 0.31 for signal RTs, as well as no main effects of order was revealed, *F*(1, 51) = 0.28, *p* = 0.60 for no-signal RTs and *F*(1, 51) = 0.51, *p* = 0.48 for signal RTs. With regard to performance errors, due to the overall low number of errors, omission and commission errors were combined and overall accuracy was submitted to the analysis. The 2 (avatar identification: *high* vs. *low*) × 2 (order: *high identification first* vs. *low identification first*) ANOVA with accuracy scores as dependent variable resulted in no significant main effects, (all *Fs* < 2.02, all *ps* > 0.16).

#### Relation between reactive response inhibition and avatar identification

To tackle our main research question and hypothesis, we further filtered the participants and included only people that showed a higher average PIS for custom as compared to generic avatars, i.e., participants who could be characterized as responders to the AIE manipulation. Specifically, delta(PIS) = custom(PIS) – generic(PIS) needed to be > 0. Forty people fulfilled this criterion; 16 played with their custom character first and 24 with the generic one first. Further, the inhibition performance advantage for custom avatars was calculated; delta(SSRT) = generic(SSRT) – custom(SSRT). Thus, since smaller SSRTs indicate a faster inhibition process, difference scores > 0 signify a performance advantage for the custom compared to the generic condition. Descriptively, participants improved over time by 21 ms (i.e., ~ 5% from 414 to 393 ms), if they played with the self-relevant avatar first, but performance got worse by 9 ms (i.e., ~ 3% from 396 to 387 ms) if they played with the generic avatar first (see Fig. 4A). In the initial analysis step, the delta(SSRT) was correlated with delta(PIS) scored for all subscales as well as with the overall mean difference. Further, since previous results showed that the order—as a changed frame of reference—is important, analysis was split by the order in which participants completed the two conditions. Results revealed that delta(SSRT) did not significantly correlate with either delta(PIS) (*r* = 0.09, *p* = 0.57), delta(PIS similarity) (*r* = 0.11, *p* = 0.50), delta(PIS embodiment) (*r* = 0.08, *p* = 0.63) or delta(PIS wishful) (*r* = 0.04, *p* = 0.83). However, order may be important given that it might change the frame of reference. Thus, correlations were re-calculated after splitting participants. If the generic avatar was used first, an identical pattern emerged with no significant correlations overall as well as for all subscales; delta(PIS) (*r* = 0.03, *p* = 0.87), delta(PIS similarity) (*r* = 0.04, *p* = 0.84), delta(PIS embodiment) (*r* = 0.15, *p* = 0.50) and delta(PIS wishful) (*r* = − 0.03, *p* = 0.88). However, if participants played with the custom avatar first, the results changed. Specifically, the correlation between delta(SSRT) and delta(PIS similarity) was significant (*r* = 0.56, *p* < 0.05), while all others were not: delta(PIS) (*r* = 0.40, *p* = 0.13), delta(PIS embodiment) (*r* = 0.17, *p* = 0.53) and delta(PIS wishful) (*r* = 0.34, *p* = 0.19). Additionally, all subscales were submitted in a stepwise manner to a regression in order to predict delta(SSRT). This procedure chooses predictors purely based on statistical merit and only significant predictors with *p* ≤ 0.05 are included stepwise and it is split by order; the regression model overall was significant only for custom avatar first task completion (*F*(1, 15) = 6.22, *p* < 0.05), with an *R*^*2*^ = 0.31 (i.e., 31% of variance explained by the significant predictors). The only significant predictor is delta(PIS similarity) (*t* = 2.50, *p* < 0.05, Cohen’s *d* = 1.34; standard model: delta(SSRT) = 0.56 * delta(PIS similarity)). For a depiction of the results and a visual reference refer to Fig. 4B.

## Discussion

We set out to investigate whether self-relevance accounts for improved response inhibition, or, more specifically, whether increased identification with a stimulus, results in improved reactive response inhibition as measured by performance in the SSG. The data indicates that customization resulted in the predicted increase in identification with the avatar thereby indicating successful manipulation. Further, if participants played with the custom avatar first, their performance increased over time and this improvement was directly associated with the degree of identification with the self-relevant avatar. This may be interpreted as an identification-dependent training effect. Specifically, it may be conjectured that the increased identification in the custom-avatar condition led to an increased commitment to the task and good task performance. In turn, this increased commitment may have enhanced the training effect only in the participants that played with their custom avatar first. Previous studies provide converging evidence for the notion that self-relevant avatars lead to an increased commitment to the task. For example, socially anxious people who identify with their own customized avatar, experience a higher degree of in-game anxiety (Dechant et al., 2021), and report a reduced, subjective workload as well as an increased feeling of gratification after a successful task completion (Qiu et al., 2021). Additionally, it should be noted that a performance increase of, on average, 22 ms corresponds to a performance increase of approximately 5%. Further, a 22 ms improvement is comparable to a performance modulation after transcranial direct current stimulation (see for example Friehs & Frings, 2018, 2019; Friehs, Brauner, et al., 2021; Friehs, Frings, et al., 2021; Friehs, Brauner, et al., 2021; Friehs, Frings, et al., 2021).

These results are in line with research demonstrating that the reference frame in which participants perform a task is crucial (Frings & Rothermund, 2017). For example, other lines of research show that the subjective perception of a stimulus as being far away from the self-results in a reduced processing speed of the stimulus (Schäfer & Frings, 2019; Schäfer et al., 2016, 2019). Thus, the perceived distance between a stimulus and the self seems to be sufficient to modulate the processing of this stimulus. Consequently, it seems plausible that in the present study, the effect emerges only for people for whom the experimental manipulation was successful (i.e., higher avatar identification for the custom compared to the generic avatar). Further, a similar effect of framing has been observed with the SSG in the initial validation of the task (), where, in short, the SSG was only rated as more enjoyable and motivating if participants were able to compare the basic and the game version with each other.

## Experiment 2: use of the self-prioritization effect

Experiment 2 set out to conceptually replicate Experiment 1 and put the effect of avatar identification on SSG performance to another test. A useful tool to create a strong association between a formerly neutral stimulus (like, e.g., an avatar in a computer game) and the self is the so-called matching paradigm (Sui et al., 2012) in which participants are instructed to associate themselves with formerly neutral stimuli. In this paradigm, to create self-associations, participants are instructed to learn associations of formerly neutral stimuli with themselves and non-self-relevant others (e.g., “You are the triangle. Someone else is the square.”). Afterward, various combinations of the used labels and stimuli are usually presented briefly on the screen and participants are instructed to decide whether each combination matches one of the previously learned associations or not (for more details, see Sui et al., 2012; Schäfer et al., 2020a, b; Schäfer & Frings, 2019). Typically and robustly, the newly self-associated stimuli are processed more efficiently as depicted by faster and more accurate responses to self-associated matching combinations (Schäfer et al., 2015; Sui et al., 2012). This effect has been demonstrated in different modalities (i.e., in vision, touch, and audition; Schäfer, et al., 2016) and has been shown with various sorts of stimuli (Frings & Wentura, 2014; Schäfer et al., 2015, 2020).

Hence, in Study 2, this paradigm was used to associate one avatar with the participant’s self and another avatar with a non-self-relevant stranger. We hypothesized the same data pattern as in Experiment 1, while this time, increased identification with the avatar should be induced via self-association. Specifically, we postulated to find enhanced SSG performance in the condition with the self-associated avatar (high-identification condition) as compared to the performance in the condition with the stranger-associated avatar (low-identification condition).

### Method

#### Sample

Participants were recruited over MTurk and 70 provided complete datasets (25 female, 45 male, mean age = 37.04, SD = 12.07). In terms of power considerations, note that the SPE in the matching task with visual stimuli was rather large in previous studies (*d*_z_ > 0.65 Schäfer et al., 2015; Sui et al., 2012; and *d* = 0.98—1.60 in Friehs, Schäfer & Frings, *under review*). However, the matching task only served as a manipulation check in our study so that we planned conservatively with a smaller effect. Completely in line with Experiment 1, a medium-sized effect of *f* = 0.35, given a medium-sized correlation between repeated performance measures of *r* = 0.4 and an α-value of 0.05, would be detected with a power of 1 – β = 0.95 in a sample of 30 participants (G*Power 3.1.3; Faul, Erdfelder, Lang, & Buchner, 2007). Again, to test for smaller effects and to deal with interaction effects with the (between-subject) factor of the order of the SSG parts, we collected data from a larger sample. Compensation schemes and consent procedures were the same as in Experiment 1.

#### Design

Like in Experiment 1, the design was a 2 (avatar identification: *high* vs. *low*) × 2 (order: *high identification* first vs. *low identification first*) mixed design with the latter factor as a between-subject factor.

#### Procedure

The procedure was in line with the procedure in Experiment 1 and the technical details of the SSG were identical. The only difference concerned the manipulation of the identification with the avatar. While in Experiment 1, identification was supposed to be increased by avatar customization, in Experiment 2, participants were associated with one avatar via instruction. Further, before the SSG, participants were asked to do the matching task and not customize their avatar.

**Fig. 5:**
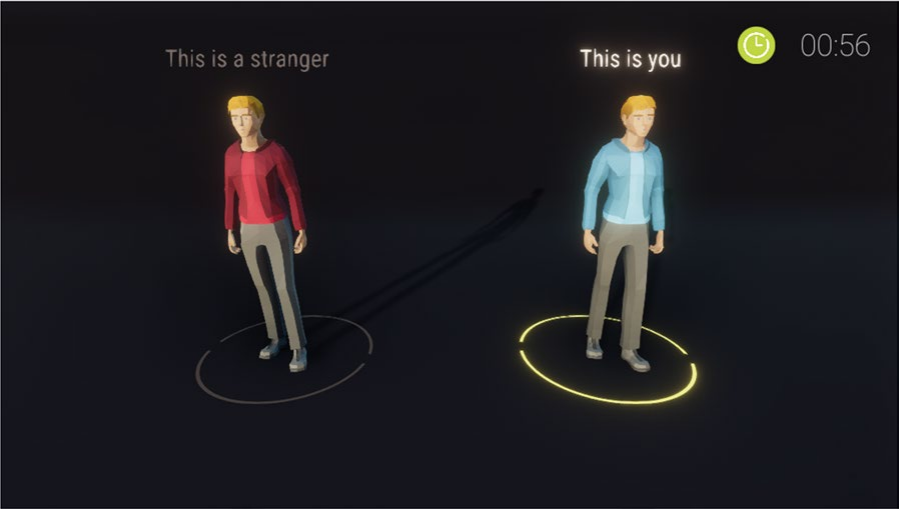
Two avatars for the matching task. The condition for the player was highlighted with the green circle and the highlighted text. In this case, the right avatar in the blue shirt would have been the avatar representing the participant in the task and the left avatar in the red shirt would have been representative of some stranger

*Matching Paradigm* We followed the typical procedure of the matching paradigm in accordance with Sui and colleagues (2012). Hence, participants were first presented with the to-be-learned assignment for 60 s (e.g., the words “I am” and “A stranger is” were presented on the screen combined with pictures of two similar, but distinguishable avatars, see Fig. 5) and were instructed to learn them by heart. The matching task followed to strengthen the self- and non-self-associations of the avatars. Additionally, the performance in the task is a manipulation check of the self- and non-self-associations. Thereto, various combinations of the labels and avatar pictures were presented on the screen and the participant had to decide whether each combination matched one of the previously learned associations or not. Each trial started with a blank screen, presented for 500 ms and followed by a fixation cross at the center of the screen for 500 ms. Then, an avatar-label combination was shown for 200 ms, followed by a black screen until the participant responded or 1500 ms had elapsed. Participants were told to place the index finger of the left hand on the S-key to indicate non-matching combinations and the index finger of the right hand on the L-key to indicate matching combinations. After each trial, participants received feedback whether their response was correct, wrong, or too slow (feedback slides were presented for 1000 ms); those trials were conducted as practice trials and were not included in the performance analysis later. After the practice trials, participants were informed that the practice phase of the matching task was over and that test trials will follow. Here, no feedback was given. The test phase consisted of 80 trials presented in random order. In detail, each label was presented in 40 trials, half of them matching and half of them non-matching pairings.

**Fig. 6:**
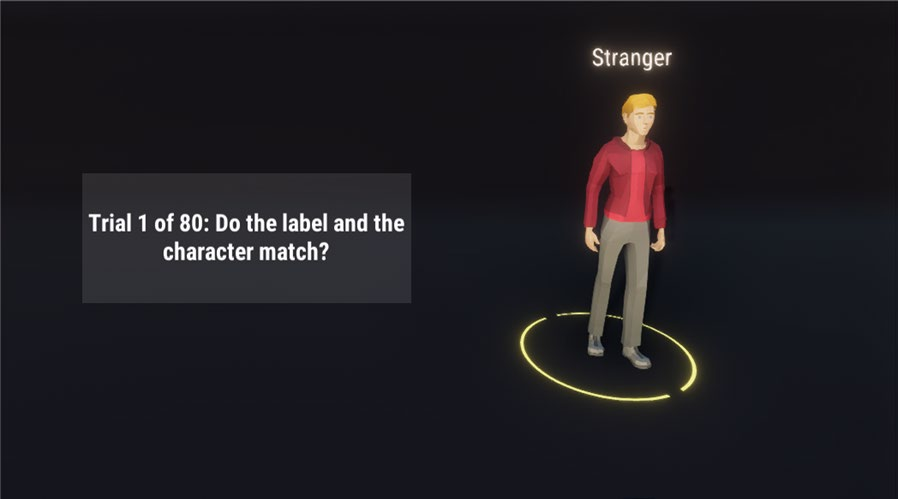
Matching Task. Each trial the participants were presented with one of the two possible avatars and a label that may or may not correspond with the depicted avatar. In this case, based on Fig. 3, the avatar depicted and the label matched

To show the combinations, a label was presented above the 3D-rendering of the avatar. With a viewing distance of about 60 cm throughout the experiment and an approximate size of 10 cm for the on-screen avatar, the labels were presented subtending 0.9° visual angle high and 1.5° width and the avatars 9.5° visual angle high and 3.8° width. We used the words “you” as the self-relevant label and the word “stranger” as the non-self-relevant stimulus. To present the avatars, a detailed 3D-render was used, which were colored either red or blue (for an example of a matching-task trial, see Fig. 6). The assignment of the colors of the avatar with the labels (either self-relevant or non-self-relevant) was balanced across participants.

#### Analysis plan

The analysis plan was identical to Experiment 1.

#### Data reduction

The procedure was identical to Experiment 1. Consequently, 19 participants had to be excluded, resulting in a final sample of 51 subjects (mean age = 38.45, SD = 12.52, 32 male and 19 female). Participants’ self-identified ethnicity was predominantly White (*n* = 38), with a minority of people identifying as Asian (*n* = 5), Black, African American (*n* = 4), or Hispanic/Latino (*n* = 3) or rather indicated two or more categories (*n* = 1).

### Results

**Fig. 7:**
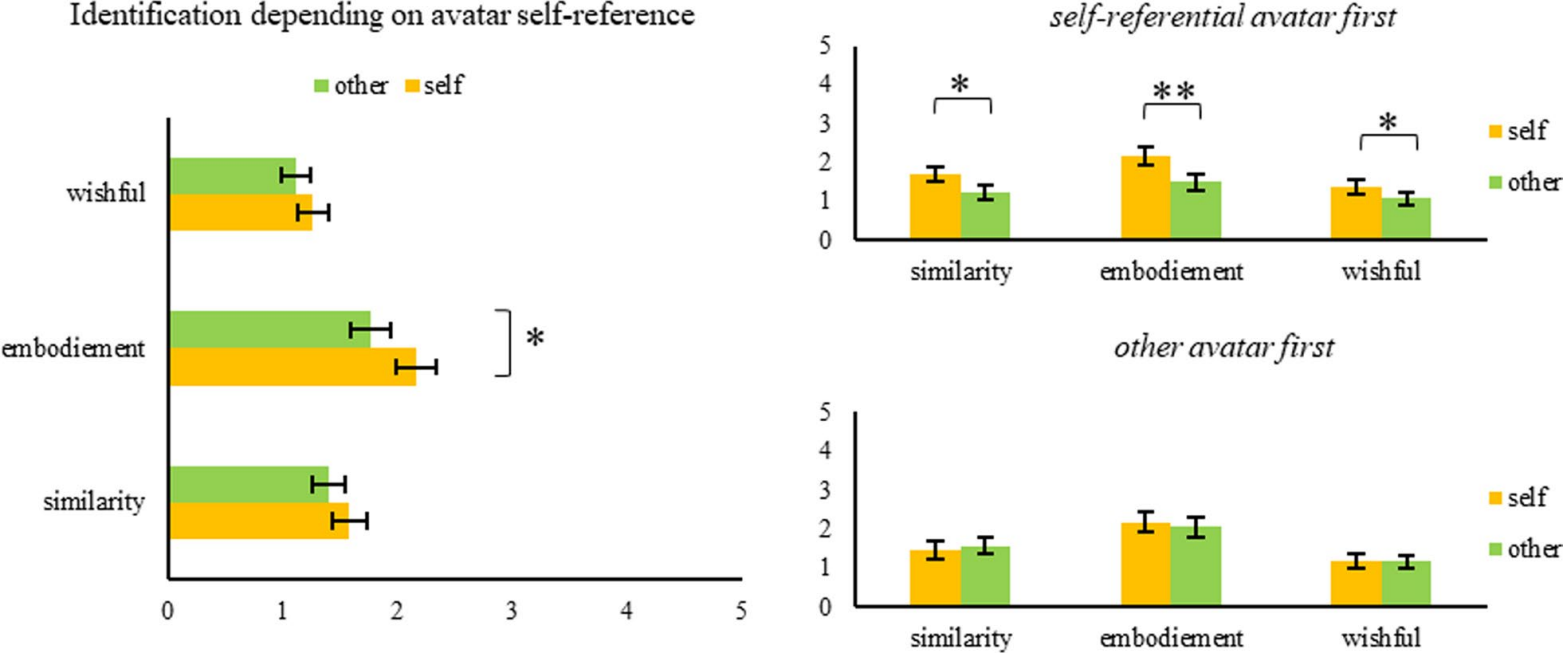
Player Identification Scale results for all participants that submitted valid data and split by the order in which the SSG was played (top right: self-referential avatar first, bottom right: other avatar first). *p < .05, **p < .001. Error bars signify the standard error of the mean

**Fig. 8:**
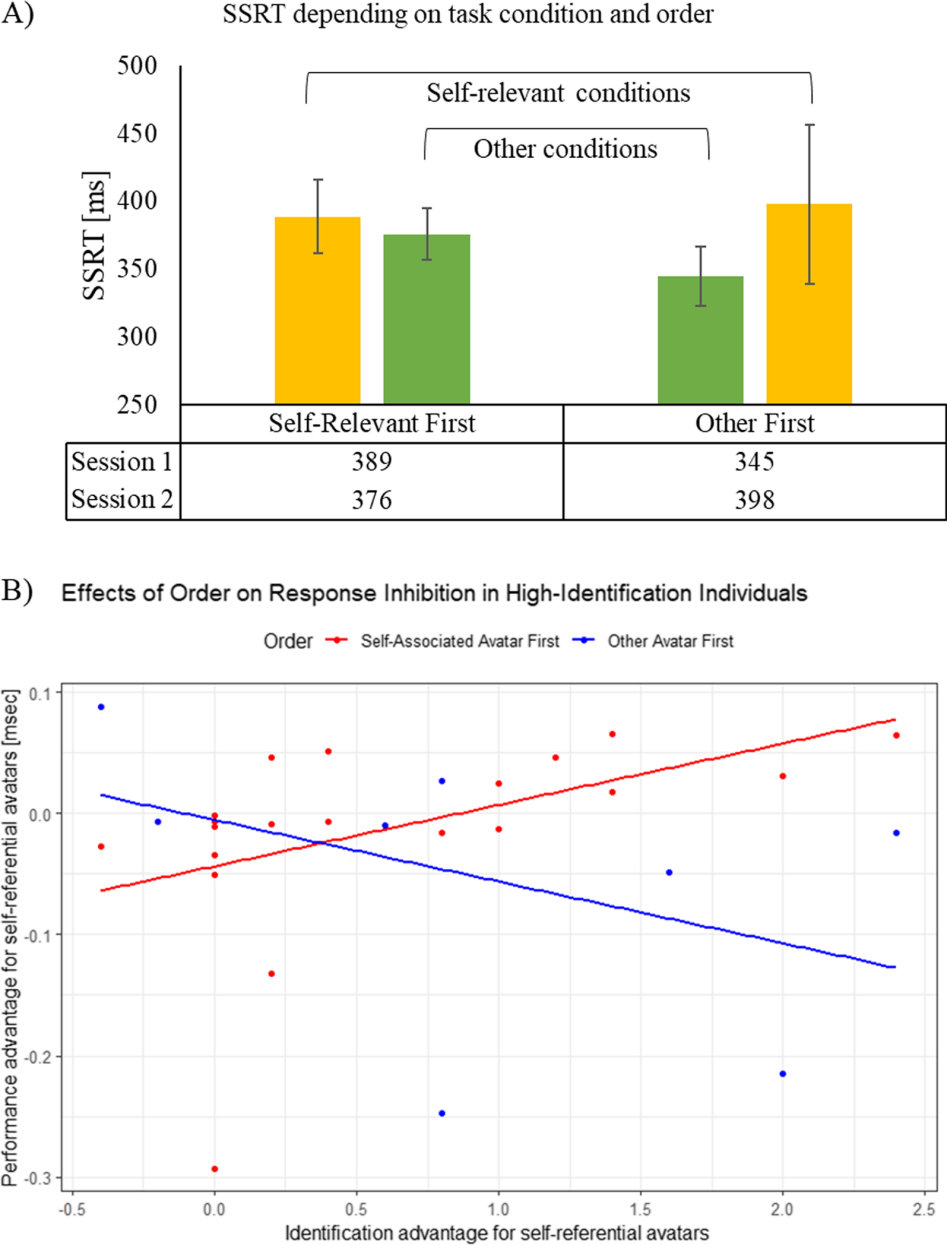
Only participants that identified with the self-relevant avatar (i.e., for whom the manipulation was successful) were considered for this analysis. Note that in the stop-signal paradigm, a lower SSRT indicates a better performance. A lower SSRT means that the estimated inhibition process is faster. **A** Descriptive statistics for both conditions depending on the task order in which they were completed. If participants played the self-relevant condition first, their SSRT (i.e., the response inhibition speed) improved from session 1–2; vice versa for the other order condition. Error bars depict the standard error of the mean. **B** Increased identification through similarity with the avatar is predictive of a performance change; as measured by the difference in SSRT between the two conditions. Self-reference was induced by the SPE, as opposed to customization as in Experiment 1. The higher identification with the self-relevant avatar, when it was used first, leads to a larger difference between the task conditions, which correspond to session 1 and 2. This performance difference is predicted by the strength of the identification with the avatar

In short, the results show that the reactive inhibition process (as measured by SSRT) is affected by the identification with the self-relevant avatar. Specifically, the practice effect was enhanced in participants with increased identification. For details on results, see Fig. 7 for the results on avatar identification and Fig. 8 for the performance change predicted by identification.

#### Manipulation check

As in Experiment 1, in Experiment 2, the PIS scores were computed and Cronbach’s alpha scores were satisfactorily high across all subscales: Similarity identification (self-relevant avatar first = 0.94, non-self-relevant avatar first = 0.92), embodied identification (self-relevant avatar first = 0.97, non-self-relevant avatar first = 0.97) and wishful identification (self-relevant avatar first = 0.93, non-self-relevant avatar first = 0.90). Hence, like in Experiment 1, player identification was measured highly reliably. A 2 (avatar identification: *high* vs. *low*) × 2 (order: *high identification first* vs. *low identification first*) × 3 (subscale: *similarity* vs. *embodiment* vs. *wishful*) MANOVA with the PIS scores as the dependent variables was calculated. The main effect of condition (*F*(1, 49) = 3.89, *p* = 0.057) only approached significance and the main effect of subscale (*F*(2, 48) = 20.89, *p* < 0.001, *η*_p_^2^ = 0.47) was statistically significant. This indicates that overall self-relevant avatars led to higher identification scores; further, the subscale similarity was rated highest across the board. The remaining main effect of order did not reach statistical significance (*F*(1, 49) = 0.14, *p* = 0.71). The two-way interaction of condition x subscale was statistically not significant (*F*(2, 48) = 2.75, *p* = 0.074), similarly, the remaining two-way interactions of condition x order (*F*(1, 49) = 3.77, *p* = 0.058) and subscale x order (*F*(2, 48) = 0.96, *p* = 0.39) were not significant. The three-way interaction was also not significant (*F*(2, 48) = 1.27, *p* = 0.29). To further explore the effects of the task conditions (self-relevant vs. non-self-relevant avatar) on player identification, follow-up *t* tests were carried out. Overall, the results reveal a generally significantly higher identification scores for only the subscale embodiment (*t*(50) = 2.69, *p* < 0.05) but not similarity (*t*(50) = 1.26, *p* = 0.2)1 or wishful identification (*t*(50) = 1.23, *p* = 0.22). Further, when order is taken into account all subscales result in statistically significant differences between the conditions but only when the self-relevant avatar is played first—similarity (*t*(25) = 3.179, *p* < 0.01), embodiment (*t*(25) = 3.62, *p* < 0.001) and wishful (*t*(25) = 2.31, *p* < 0.05)—which reveals the importance of task order. If participants played with the non-self-relevant avatar first, none of the three subscale comparisons reached significance: similarity (*t*(24) =  − 0.43, *p* = 0.67), embodiment (*t*(24) = 0.49, *p* = 0.63), and wishful identification (*t*(24) = 0.00, *p* = 1.00). These results confirm that the experimental manipulation was effective.

#### Self-prioritization effect

Only correct responses that were faster than 200 ms and slower than 3 interquartile ranges above the third quartile of the overall correct RT distribution were used for RT analysis. A 2 (trial type: *matching* vs. *non-matching*) × 2 (identity: *self* vs. *other*) ANOVA revealed a significant main effect of trial type for both dependent variables, the *F*(1, 50) = 23.80, *p* < 0.001, η_p_^2^ = 0.32 for RTs and *F*(1, 50) = 4.09, *p* < 0.05, η_p_^2^ = 0.08 error rates, indicating better performance in matching trials. Also the main effect of identity was significant for both variables, *F*(1, 50) = 73.53, *p* < 0.001, η_p_^2^ = 0.60 for RTs and *F*(1, 50) = 10.98, *p* < 0.01, η_p_^2^ = 0.18 for error rates, indicating that the association had an influence on subsequent responses. As typical in the paradigm, the two-way interaction was also significant, *F*(1, 50) = 28.75, *p* < 0.001, η_p_^2^ = 0.37 for RTs and *F*(1, 50) = 12.11, *p* < 0.001, η_p_^2^ = 0.20 for error rates, which simply indicates that the effect of the association works differently in matching and non-matching trials. The SPE was computed as the difference between RTs/error rates in self-related matching trials and RTs/error rates in other-related matching trials. The data revealed a significant SPE in both dependent variables, (*t*(50) = 8.96, *p* < 0.001), for RTs and (*t*(50) = 4.01, *p* < 0.001), for error rates. In sum, this result indicated a significant prioritization of the self-related combination against the other-related combination and thereby indicates that the association of one avatar with the participant’s self, worked successfully.

#### Preliminary SSG analysis

For details on the validation, please refer to Study 1. The analysis revealed that signal-response time and no-signal RT are significantly different as indicated by the significant main effect trial type (*F*(1, 50) = 263.86, *p* < 0.001, *η*_p_^2^ = 0.84). The main effect condition (*F*(1, 50) = 0.21, *p* = 0.65) as well as the interaction (*F*(1, 50) = 0.01, *p* = 0.91) did not reach statistical significance. These results validate the gathered behavioral data.

#### Overall performance analysis

SSRT as an indicator of the speed of the reactive response inhibition process was submitted to an avatar identification: 2 (*high* vs. *low*) × 2 (order: *high identification first* vs. *low identification first*) ANOVA. All effects were statistically non-significant: main effect condition (*F*(1, 49) = 2.81, *p* = 0.10), main effect order (*F*(1, 49) = 1.38, *p* = 0.25) and the interaction (*F*(1, 49) = 0.96, *p* = 0.33). The same analysis was carried out with SSD as the dependent variable. It revealed two non-significant main effects of condition (*F*(1, 49) = 0.96, *p* = 0.33) and order (*F*(1, 49) = 0.11, *p* = 0.74), while the interaction (*F*(1, 49) = 10.68, *p* < 0.01, *η*_p_^2^ = 0.18) was statistically significant, which indicates a practice effect from session 1 to 2. Due to the overall low number of errors, omission and commission errors were combined and overall accuracy was submitted to the analysis. The 2 (*high* vs. *low*) × 2 (order: *high identification first* vs. *low identification first*) ANOVA resulted in no significant main effects of condition (*F*(1, 49) = 0.65, *p* = 0.42), order (*F*(1, 49) = 0.55, *p* = 0.46) or the interaction (*F*(1, 49) = 3.47, *p* = 0.07). Further, both no-signal reaction times (i.e., correct reaction speed during go trials) and signal reaction times (i.e., the speed of false reactions during a stop trial) were submitted to the same analysis and both analyses revealed the same pattern of results. In detail, there were no main effects of condition (no-signal RT: *F*(1, 49) = 0.25, *p* = 0.62 and signal RT: *F*(1, 49) = 0.16, *p* = 0.70), no main effects of order (no-signal RT: *F*(1, 49) = 0.67, *p* = 0.42 and signal RT: *F*(1, 49) = 0.73, *p* = 0.40), but a significant interaction (no-signal RT: *F*(1, 49) = 17.54, *p* < 0.001, *η*_p_^2^ = 0.26 and signal RT: *F*(1, 49) = 17.64, *p* < 0.001, *η*_p_^2^ = 0.27). This indicates a practice effect from session 1 to 2.

#### Relation between reactive response inhibition and avatar identification

To tackle our main research question and hypothesis, we further filtered the participants and only people that showed a higher average PIS for self-relevant as compared to non-self-relevant avatars were included, representing participants who were responders to the SPE manipulation. Specifically, delta(PIS) = self-relevant(PIS) – non-self-relevant(PIS) needed to be > 0. Twenty-eight people fulfilled this criterion; 20 played with their self-relevant character first and eight with the non-self-relevant one first. This further indicates that order is important. The difference scores of delta(SSRT) and delta(PIS)—including all subscales—were calculated the same as in Study 1 and analysis followed the same procedure. Overall results revealed that delta(SSRT) did not significantly correlate with either delta(PIS) (*r* = 0.21, *p* = 0.29), delta(PIS similarity) (*r* = 0.06, *p* = 0.77) or delta(PIS embodiment) (*r* = 0.12, *p* = 0.54). But a significant correlation with delta(PIS wishful) (*r* = 0.41, *p* < 0.05) emerged. However, previous results suggest that the order is important given that it might change the frame of reference. Descriptively, participants improved over time by 13 ms (i.e., ~ 4% from 389 to 376 ms) if they played with the self-relevant avatar first, but their performance worsened by 53 ms (i.e., ~ 13% from 345 to 398 ms) if they played with the generic avatar first. See Fig. 8A. Further, correlations were re-calculated after splitting participants. If the non-self-relevant avatar was used first, no correlations were significant: delta(PIS) (*r* = − 0.12, *p* = 0.77), delta(PIS similarity) (*r* = − 0.43, *p* = 0.28), delta(PIS embodiment) (*r* = − 0.28, *p* = 0.50) and delta(PIS wishful) (*r* = − 0.68, *p* = 0.07). However, if participants played with the self-relevant avatar first, the results changed. Specifically, the correlation between delta(SSRT) and delta(PIS similarity) was significant (*r* = 0.48, *p* < 0.05) and so was the correlation with delta(PIS) (*r* = 0.48, *p* < 0.05), while all others were not: delta(PIS embodiment) (*r* = 0.42, *p* = 0.66) and delta(PIS wishful) (*r* = 0.41, *p* = 0.71). Additionally, all subscales were submitted in a stepwise manner to a regression in order to predict delta(SSRT). This procedure chooses predictors purely based on statistical merit and only significant predictors with *p* ≤ 0.05 are included. The regression model overall was only significant when participants completed the self-relevant avatar task first (*F(*1, 19) = 5.33, *p* < 0.05), with an *R*^*2*^ = 0.23 (i.e., 23% of variance explained by the significant predictors). The only significant predictor is delta(PIS similarity) (*t* = 2.31, *p* < 0.05, Cohen’s *d* = 1.18; standard model: delta(SSRT) = 0.48 * delta(PIS similarity)). For a depiction of the results and a visual reference, refer to Fig. 8B.

## Discussion

Like Experiment 1, Experiment 2 was carried out to investigate whether self-relevance, as induced by the SPE, can improve response inhibition. The two experiments differ with regard to how self-relevance is induced. The data from Experiment 2 reveals that the manipulation was successful, and the SPE induced higher identification with the self-labeled avatar. Further, order played a significant role in that an effect of avatar identification was only given when participants experienced the self-relevant (i.e., high identification) avatar first. Specifically, the higher identification with the custom avatar was predictive of the performance improvement from session 1 to session 2, only if participants played with the self-relevant avatar first. Importantly, this improvement was only present in participants that identified with the avatar that was labeled self. On average, participants improved by 13 ms, which is an improvement in inhibitory performance similar to that of smaller performance changes induced by transcranial direct current stimulation (see for example Friehs & Frings, 2018, 2019; Friehs, Brauner, et al., 2021; Friehs, Frings, et al., 2021; Friehs, Brauner, et al., 2021; Friehs, Frings, et al., 2021). Again, as in Experiment 1, this could be seen as evidence that the increased identification enhances the commitment to the task, which in turn benefits training. In agreement with Experiment 1, this further supports the notion that subjective evaluation of an avatar is important and that this in turn may modulate performance.

Nevertheless, there was an important difference between the results of both studies. The effect of order on the PIS scores were more pronounced in Experiment 2 compared to Experiment 1: participants were much more likely to identify with the self-relevant avatar, if that avatar was played with first and not second. Thus, the identification induced by the SPE does not seem to be as long-lasting as the identification induced by customization; at least when identification is measured with the PIS. One reason for the longer-lasting effect of customization may be choice and effort put into the choice, which would be in line with past research suggesting that choice increases immersion into the virtual world (Bowey, Friehs, & Mandryk, 2019).

## General discussion

Overall, the results from both experiments show that a subjectively self-relevant avatar may facilitate a performance improvement over time. This may be because of an increased commitment to and investment in the task at hand. Self-relevance was induced via two different pathways—the AIE and SPE—but the results replicated each other.

### Theoretical implications

The results provide evidence that self-relevance modifies performance. Based on the results and positive manipulation checks, it seems plausible to assume that the intrinsic motivation to perform the task as best as possible was manipulated in both experiments. This assumption is in line with previous results implying that increased avatar identification can facilitate a higher degree of in-game anxiety (Dechant et al., 2021), and a reduced, subjective workload as well as an increased feeling of gratification after a successful task completion (Qiu et al., 2021).

**Fig. 9:**
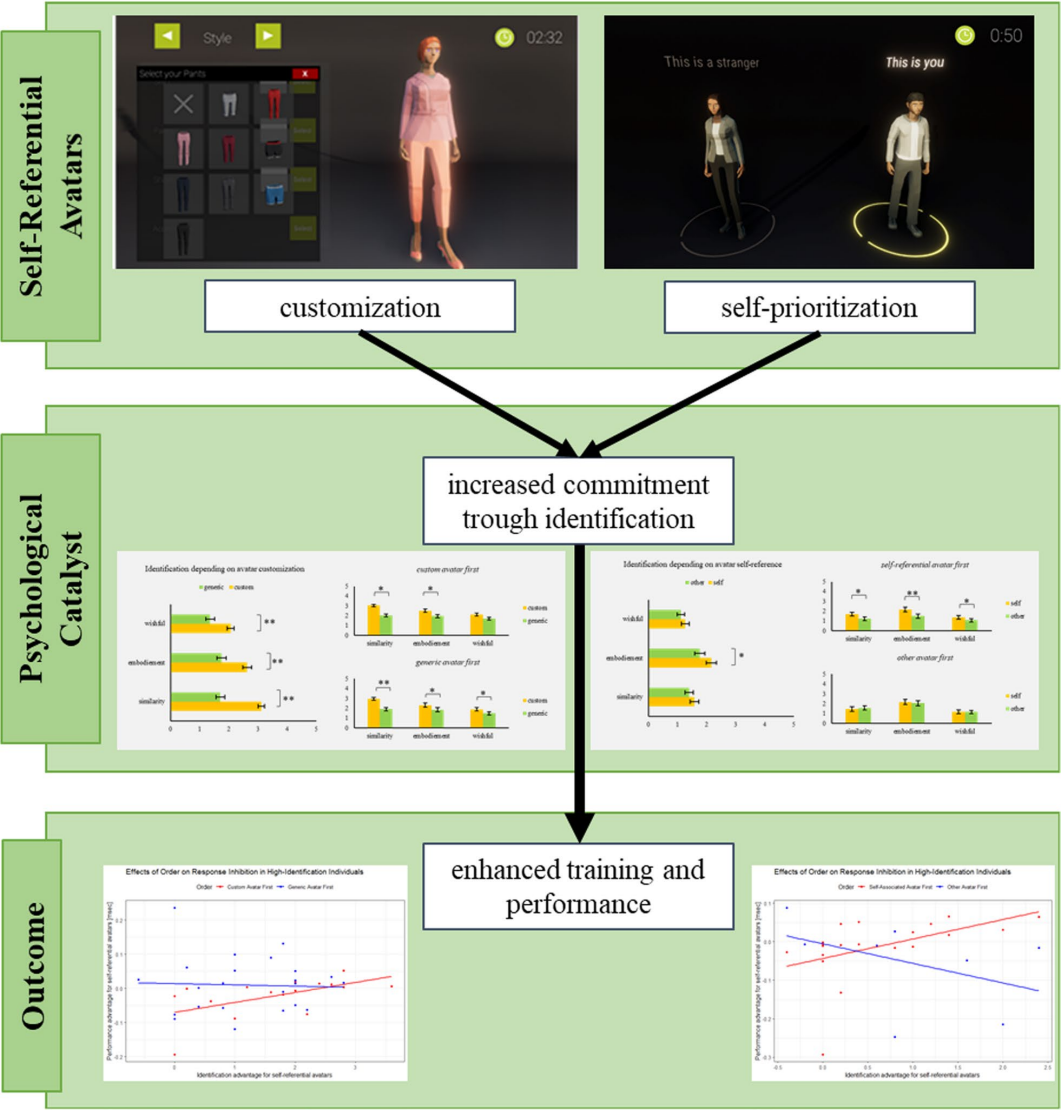
Summary of the results and the proposed underlying explanation for them. In short, self-referential avatars are hypothesized to increase commitment to the task through identification and in turn enhance the performance

Furthermore, the dependence of the results on the order in which the different conditions (self-relevant vs. non-self-relevant) were experienced provides insight into the initial fragility of the avatar identification. Even though overall the results show on average a higher identification with the self-relevant avatar, the difference in identification is largest if participants play with the self-relevant avatar first. This may be due to a framing effect; in that the first avatar sets the frame of reference and participants will identify with the first avatar that represents them in the task and more identification with the second avatar as compared to the first one is unlikely. Conversely, if participants play with their own avatar first, the discounting of the second avatar in relation to the first one is more likely. See Fig. 9 for a model summary. With regard to the facets of identification that are responsible, the results show that in both experiments, the subscale similarity of the PIS is the best predictor of performance. While that intuitively is plausible for Experiment 1—after all, participants are tasked with an avatar that represents themselves in the task—in Experiment 2, it would have been potentially more likely that embodied identification might produce the strongest effects. However, when the specific questions for those subscales are considered, the similarity subscale should arguably result in the strongest effect (see Methods sections for scale details).

However, it is unclear if the computed differences scores are due to lower motivation for the subsequent not-self-relevant avatar or increased motivation for the self-relevant avatar condition. The present data cannot answer this question conclusively, but it seems plausible that the difference in motivation for play with the self-relevant and not-self-relevant avatar is crucial. This line of thinking is supported by the correlation between the strength of the identification and the increased performance over time. Further, the lower motivation for the second session plays a bigger role, when participants play with the generic avatar first. Since participants in the self-relevant avatar condition improve their performance over time and show better performance in the generic avatar condition, the effect of decreased motivation cannot be strong. This may also indicate a potential decay of the identification effect.

The present results are closely related to a study by Golubickis and colleagues (Golubickis et al., 2021). In their study, the authors had participants performing a stop-signal task, in which they had to react to targets that either were labeled as belonging to themselves or another person. Their results show an increase in performance for self-owned items, as indicated by lower SSRTs. The authors interpret this as evidence for a beneficial effect of self-relevance on response inhibition. However, the present studies, but specifically Experiment 2, are somewhat different from Golubickis et al. First, in Experiment 2, prior to the response inhibition test, participants had to perform a matching task, which both served as a reinforcement of the self vs. other distinction as well as a manipulation check. Second, while Golubickis et al., (2021) directly manipulated the target self-relevance, in Experiment 2, the self-relevance of a non-relevant stimulus was manipulated. Third, in both experiments reported in this manuscript, participants were tasked to directly identify with an avatar in the task context. In contrast to this, in Golubickis et al., (2021), participants do not identify with an object (in their case pens or pencils) in the task itself, but are merely told to represent an object as being owned by themselves. Consequently, the self-relevance induced in both experiments of the present manuscript was more ecologically valid and one could argue that if participants were to be tasked with reacting to a self-relevant avatar (i.e., the avatar as the target), an even greater effect as compared to Golubickis et al., would be observed. Taken together, although both studies may seem similar at first glance, there are several important differences and thus, it is still uncertain to which extent self-relevance may influence response inhibition.

Further, self-relevance effects may have been studied inadvertently by other researchers before. For example, there is some evidence to suggest that threatening and thereby arousing stimuli (Pessoa et al., 2012; Verbruggen & De Houwer, 2007) as well as monetary rewards (Boehler et al., 2012, 2014; Herrera et al., 2014) can modulate response inhibition capabilities. With those studies highlighting the effect of arousal, one might postulate that internal aspects (because of monetary reward) rather than external aspects were causal for changes in response inhibition. One possible theoretical explanation for these effects are that threatening stimuli are not only more arousing but also inherently self-relevant and thus draw upon attentional resources. Hence, previous studies concluded that an emotional character of stimuli can either enhance or impair cognitive performance depending on the *emotional potency* of the stimuli involved and the way an inappropriate response can be inhibited, depends on *how much* one is interrupted from focusing on not doing it. Put differently, the difference between the effects of the high- and low-threat stimuli on response inhibition might itself be explained by the difference in the stimuli’s self-relevance.

Additionally, self-relevance could potentially help to explain the inconsistent effects of stress on response inhibition (for a meta-analysis of stress effects see Shields et al., 2016). Thus, if self-relevance modulates response inhibition, then only stress that is perceived as a threat to the core self-concept changes behavior. And specifically, only in tasks that the participant of the study deems to be self-relevant. This self-relevance could be facilitated with the use of effects like the SPE and AIE. One stress paradigm that arguably makes use of this self-relevance effect is the virtual reality version of the Trier Social Stress Test, in which participants see themselves virtually in front of a judging panel as if they were in the virtual space themselves (Zimmer, Buttlar, et al., 2019; Zimmer, Wu, et al., 2019; Zimmer, Wu, & Domes, 2019). However, in setups in which a virtual reality experiment is not possible, avatar identification through different means (i.e., the AIE and SPE) could be used to increase potential effects of stress on performance (see also Dechant et al., 2021).

### Limitations

Although we applied the same analysis to both conceptually similar studies and replicated the results across both, there are some limitations to consider. First, only a portion of participants showed a larger identification score in the self-relevant conditions compared to the not self-relevant one (69% in Experiment 1 and 55% in Experiment 2). One may argue that the effect was slightly weaker in Experiment 2 compared to Experiment 1, but it should also be considered that in Experiment 2, the crucial manipulation of self-relevance can be statistically tested and the SPE was significant. Second, at first glance the overall effect of identification on performance may seem small, given the boundary condition of a successful manipulation has to be met. However, this may also be partially a ceiling effect since only healthy adults participated in the study and the amount of influence an avatar identification in one session may exert is limited compared to other methods such as noninvasive brain stimulation that strongly and directly manipulate the underlying neural activity. Nevertheless, the obtained effect sizes for the regression are large with Cohen’s *d* = 1.34 and 1.18 in Experiment 1 and 2, respectively. Third, in Experiment 2, the avatars that we used were non-binary with regard to their bodily proportions, and thus, the avatars used were not necessarily reflective of the individual that was performing the task. But given that the data replicated the previous results, the effect seems sufficiently robust. Fourth, although this was a proof-of-concept study, and we were open to be guided by the data pattern, we expected that a straightforward comparison of task conditions would reveal a significant difference. However, the data exposed an influence of interindividual differences and considerable order effects. It may have been possible that the act of taking away the self-relevant avatar after the initial introduction of it, could have led to a decrease in motivation. However, this may be unlikely, since the task instructions made it clear multiple times that participants needed to play the game with both avatars. Thus, there was never a promise of getting to play with the self-relevant avatar first. Alternatively, a decay of identification with the avatar after some time could be assumed; similar to a once learned association needs to be reinforced to be learned. As these strong order effects were unexpected, future research is needed to explain why certain individuals identify with a digital avatar more easily.

### Application

This project bears implications for everyday life, game design, and therapy alike. Increasing avatar identification and self-relevance might be simple ways to increase a training effect and fuel positive therapeutic outcomes. Past research suggests that response inhibition capabilities can be improved by training (Kramer et al., 1999; Wang et al., 2013) and there are established gaming-related frameworks that encourage spaced practice and repeated engagement with the task over a sustained period of time (Alexandrovsky et al., 2019, 2021; Johanson et al., 2017, 2019; Piller et al., 2020). As the present study utilizes a gamified version of a task and previous studies showed response inhibition enhancements after training, it seems reasonable to assume that this also applies to an esports setting or gaming in general. For example, in competitive games, such as League of Legends by RiotGames, players may choose one out of hundreds of available heroes to play with and compete with an enemy team. Players need to react quickly to the environment to gain an advantage over the enemy; for example, players may need to stop their movement to avoid an incoming projectile attack. The present results could be applied to this situation, suggesting that maybe the identification with the avatar may be advantageous for performance. However, in contrast to esports athletes that may be able to pick and choose their avatar as they please, the average gamer may not have this opportunity. Avatar customizations, at least cosmetic ones, are often locked behind a paywall and since they do not provide a power-boost for the character in game, they are often assumed to not matter. While this study cannot provide conclusive proof to claim otherwise, the results do suggest that being able to express yourself in game and identifying with the avatar may be important for performance. Identification with the avatar, especially through similarity to the avatar, may be particularly difficult for minority groups that are underrepresented in games. Nevertheless, it should be mentioned that the present studies suggest that both identification through customization as well as assigned self-relevance may affect commitment. How both of those effects interact and if there may be an (over)additive effect on performance or how long the effect holds, is a topic for future research.

### Future research

First, the underlying mechanism of action should be investigated. It seems plausible that self-relevance fostered the investment and commitment to the task, but this mechanism cannot be concluded based on the present results. Second, based on the present results, training studies could be carried out that test participants performance over multiple sessions and days. Based on the findings from the two experiments, training with a self-relevant avatar may enhance the overall training effect. Third, it remains unclear how long the effects of avatar identification last. Potentially, a re-identification phase would be needed regularly, if a multi-day study would be carried out. Fourth, in theory both the SPE and AIE can be combined in one study and potentially contribute to an (over)additive effect. In detail, the matching paradigm outlined in Experiment 2 could be implemented after the customization described in Experiment 1. Fifth, this paradigm could be transferred to more applied settings as outlined in the paragraph above. Sixth, if participants can identify with an avatar that represents themselves, it may also be possible for them to identify with avatar that carries certain traits and characteristics. For example, if an avatar is described as slow, and participants are told to identify with the avatar, it may be possible that their own reaction time is slowed down in accordance with the character trait.

### Conclusion

Taken together, our results suggest that avatar identification, as induced by the AIE and SPE, can enhance performance. Specifically, the increased identification was predictive of an increase in response inhibition speed, if participants played with the self-relevant avatar first. Importantly, both experiments detailed in this manuscript are conceptual replications of each other and produce comparable results. 

## Data Availability

All data can be accessed free of charge on the OpenScienceFramework (https://osf.io/twfa3/). Study material (i.e., the stop-signal game) can be provided by the authors upon reasonable request.
